# Visualization of Stress Fiber Formation Induced by Photodynamic Therapy with Porphylipoprotein

**DOI:** 10.3390/nano14231862

**Published:** 2024-11-21

**Authors:** Atsushi Taninaka, Hiromi Kurokawa, Mayuka Kamiyanagi, Osamu Takeuchi, Hirofumi Matsui, Hidemi Shigekawa

**Affiliations:** 1 Institute of Pure and Applied Sciences, University of Tsukuba, 1-1-1 Tennodai, Tsukuba 305-8573, Japan; jun_t@bk.tsukuba.ac.jp (A.T.); kami.m.311007@gmail.com (M.K.); takeuchi.osamu.ft@u.tsukuba.ac.jp (O.T.); 2 TAKANO Co., Ltd., Miyada-mura, Kamiina-gun, Nagano 399-4301, Japan; 3 Faculty of Medicine, University of Tsukuba, 1-1-1 Tennodai, Tsukuba 305-8575, Japan; hkurokawa.tt@md.tsukuba.ac.jp (H.K.); hmatsui@md.tsukuba.ac.jp (H.M.); 4 Phycochemy Co., c/o ABES, University of Tsukuba, 1-1-1 Tennodai, Tsukuba 305-8573, Japan

**Keywords:** photodynamic therapy (PDT), porphylipoprotein (PLP), autophagy, atomic force microscopy (AFM)

## Abstract

We investigated stress fiber formation induced by photodynamic therapy (PDT) with porphylipoprotein (PLP) by observing actin filaments by super-resolution confocal microscopy and measuring the cellular elastic modulus by atomic force microscopy. We identified different intracellular mechanisms of stress fiber formation between RGM1 epithelial cells, which were derived from rat gastric mucosa, and RGK1 cells, which were cancer-like mutants of RGM1. Our findings show that when PLP is used as a photosensitizer in PDT, it selectively induces necrosis in tumors with minimal impact on the surrounding normal tissues, as it is less likely to cause blood flow obstruction.

## 1. Introduction

Recent advances in drug discovery have shifted focus from simply designing highly effective molecules to engineering multifunctional nanomaterials. These nanomaterials integrate various components, such as inorganic materials (e.g., quantum dots, metal nanoparticles, magnetic nanoparticles, porous silica) and biocompatible organic materials (e.g., phospholipids or hydrophilic functional groups). By combining these components, researchers have developed composite nanoparticles with enhanced capabilities, such as targeted drug delivery and tumor-specific actions [1,2,3,4,5,6,7]. These nanoparticles utilize mechanisms like the Enhanced Permeability and Retention (EPR) effect to accumulate around cancer tissues, enabling their use for both therapeutic applications, such as lesion destruction, and diagnostic purposes [8,9].

However, conventional materials such as polyethylene glycol (PEG), while widely used to improve biocompatibility and stability, have faced challenges including potential immunogenicity and limited biodegradability. Porphylipoprotein (PLP) is a nanoparticulate substance recently developed with high biocompatibility and tumor selectivity [7,10,11]. PLP is also a drug delivery system characterized by its capability to encapsulate diverse molecules. Additionally, PLP has been studied as a highly effective photo-sensitizer in photodynamic therapy (PDT) and photodynamic diagnosis (PDD). PDT is a treatment strategy by which a photosensitizer is administered, followed by light irradiation at a specific wavelength to trigger a photochemical reaction, generating reactive oxygen species (ROS) that damage tumor cells. PDD, on the other hand, is a diagnostic method that uses a photosensitizer selectively taken up by tumors. Upon irradiation, the photosensitizer emits fluorescence, helping in detecting tumors that are otherwise difficult to identify. Furthermore, PDT with PLP has recently demonstrated its abscopal effect, which suppresses tumors at distant sites [12].

Recent studies have also revealed new biological effects of PLP that extend beyond its role in ROS generation. For instance, we demonstrated in our previous study that PLP induces autophagy, thereby producing novel therapeutic effects.

**Figure 1:**
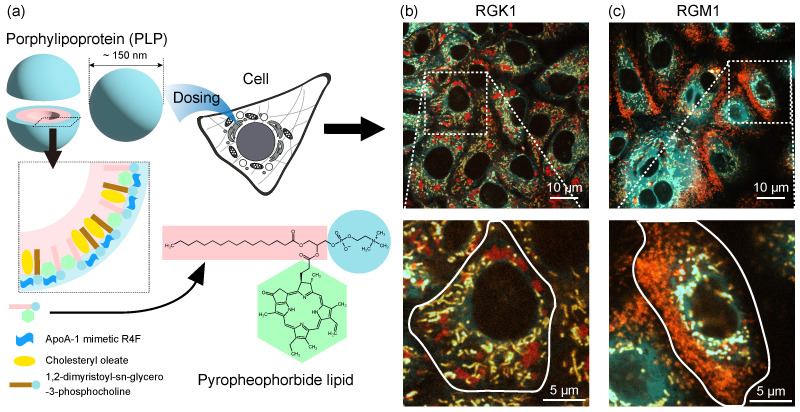
Overview of porphylipoprotein (PLP); (**a**) porphyrin-based ultrasmall nanostructure that mimics lipoproteins, which are complexes of lipids and proteins. It has a core–shell structure where the outer part is composed of hydrophilic functional groups and hydrophilic molecules, whereas the inner part is composed of hydrophobic functional groups and hydrophobic molecules [7,10,11]. Fluorescence images of RGK1 (**b**) and RGM1 (**c**) observed by super-resolution microscopy. The images show that the endoplasmic reticulum (ER) fluorescence (pseudo-colored cyan), mitochondrial fluorescence (pseudo-colored orange), and PLP fluorescence (pseudo-colored red) merged. Enlarged images of the areas enclosed in white dashed lines are shown below each, in which a single cell is outlined with a white solid line [13].

The structure of PLP, used in PDT, is shown in Figure 1a. PLP is an ultra-small, porphyrin-based nanostructure that mimics lipoproteins, complexes of lipids and proteins, with a core–shell architecture. The outer layer consists of hydrophilic functional groups and molecules, whereas the inner core is composed of hydrophobic functional groups and molecules [10,11,12]. The pyropheophorbide–lipid conjugate that constitutes PLP includes a phospholipid and a chlorin ring. The hydrophobic chlorin ring is arranged inside the particle, forming a spherical structure.

In PLP-based PDT, as with conventional PDT, PLP is administered to a lesion, and light irradiation generates ROS to destroy the lesion, producing a therapeutic effect [7,10,11,12,13,14,15]. Interestingly, our recent study demonstrated that the efficacy of PDT depends on the organelle where PLP accumulates, as this determines the target of ROS and influences the required irradiation conditions [13]. This observation highlights the potential for organelle-specific localization to enhance PDT precision and reduce side effects.

To further explore the intracellular dynamics of PLP and its role in autophagy, we examined its localization within organelles in cancer-like RGK1 cells and normal epithelial RGM1 cells using fluorescence imaging (Figure 1b,c), which were obtained by the same method used in the previous work. Here, RGM1 cells represent normal epithelial cells derived from rat gastric mucosa, while RGK1 cells are cancer-like variants of RGM1.

In RGK1 cells, phagosomes are located around the endoplasmic reticulum (ER) and mitochondria, near the nucleus [13]. In contrast, in RGM1 cells, phagosomes are largely absent from these regions and are instead positioned at the cell periphery. This distribution pattern reflects different autophagy mechanisms [16,17,18,19,20,21,22]. In RGK1 cells, phagosomes containing PLP migrate toward the nucleus along the autophagy pathway. This allows nutrient-deprived cancer cells to replenish resources by eventually fusing with lysosomes to form phagolysosomes. In contrast, in RGM1 cells, phagosomes with PLP migrate to the cell periphery, driven by the self-cleansing function of autophagy.

**Figure 2:**
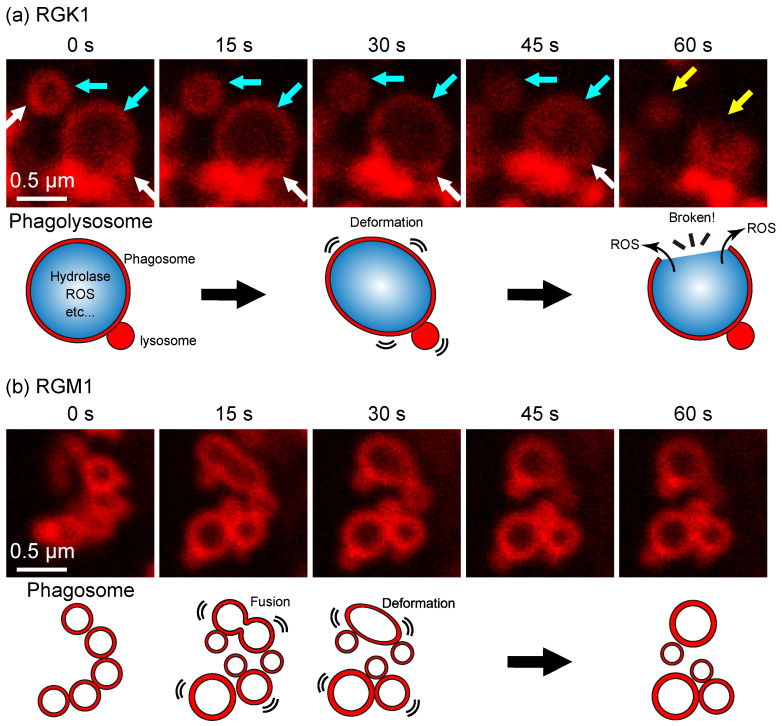
Fluorescence time-lapse images of phagosomes in (**a**) RGK1 and (**b**) RGM1 cells under 1 min light irradiation, and a schematic diagram of the behavior of phagosomes under oxidative stress induced by PDT. In (**a**), the blue, white, and yellow arrows indicate the phagosome, lysosome, and phagolysosome destroyed by PDT, respectively [15].

It was also found that phagosome size differs between RGK1 and RGM1 cells [13]; in particular, the response of phagosomes to oxidative stress shows significant differences between these cells [13]. Figure 2 shows fluorescence images of phagosomes under oxidative stress in RGK1 and RGM1 cells during 1 min light irradiation, along with a schematic of phagosome behavior. In RGK1 cells, phagolysosomes (formed by phagosome–lysosome fusion) are destroyed by PDT, whereas in RGM1 cells, phagosomes fuse with each other, resulting in observable size changes [13].

As noted, phagosomes containing PLP follow the autophagy pathway, fusing with lysosomes to form phagolysosomes that are typically degraded by internal hydrolases. Figure 2 shows that when PDT is applied after PLP accumulation and phagolysosome formation in RGK1 cells, the phagosomal membrane is disrupted by ROS generated by light irradiation, leading to the leakage of hydrolases and ROS into the cells [13]. In contrast, in RGM1 cells, peripherally localized phagosomes undergo fusion and size changes under PDT but remain intact [13]. These findings suggest that this process underlies the stronger effect of PLP-based PDT on cancer cells [13,15].

Another key therapeutic mechanism of PDT, alongside its direct ROS-induced necrotizing effect on cancer cells, is the vascular shutdown effect [23,24,25,26,27,28,29]. Here, we focus on this effect. The vascular shutdown effect occurs as ROS generated by PDT activate RhoA, promoting actin filament production and myosin light chain phosphorylation, which leads to increased stress fiber formation [23,24,25,26,27,28,29]. However, in PLP-based PDT, RhoA activation is delayed in RGK1 cells, not occurring in the first minute during phagolysosome destruction [14]. Once phagolysosomes are destroyed and ROS start leaking, actin filament production and myosin light chain phosphorylation increase rapidly, leading to enhanced stress fiber formation [13,14]. This indicates that the vascular shutdown effect is promoted by ROS leaking from phagolysosomes. In contrast, in RGM1 cells, where phagosomes are not destroyed, it remains unclear whether the vascular shutdown effect does not occur if singlet oxygen from PLP fails to induce this effect (i.e., stress fiber formation is not triggered), or at which stage the mechanism might activate.

To fully harness the effects of PDT using PLP, a thorough understanding of the underlying mechanisms is essential. In this study, we aimed to elucidate different intracellular mechanisms of stress fiber formation between RGK1 and RGM1 cells by observing actin filaments by super-resolution confocal microscopy and measuring cell elasticity by atomic force microscopy (AFM).

## 2. Materials and Methods

**Preparation of PLP.** PLP is a photosensitizer that contains a hydrophobic drug-loadable core enveloped in a porphyrin–lipid monolayer and constrained by ApoA-1 mimetic R4F (Ac-FAEKFKEAVKDYFAKFWD) peptide networks [11,13,14,15].

Cell culture. The rat gastric epithelial cell line RGM1 was purchased from RIKEN CELLBANK (Tsukuba, Japan) [30]. The RGK1 cells used in this study were rat gastric mucosa-derived cancer-like mutant cells [30], which are chemically induced oncogenic cancer-like mutant cells of RGM1. RGK1 cells were cultured in DMEM/F12 without L-glutamine (Sigma-Aldrich^®^ Merck KGaA, Darmstadt, Germany). RGM1 cells were cultured in DMEM/F12 with L-glutamine (Gibco™, Thermo Fisher Scientific Inc., MA, USA).

**Sample preparation.** PLP (final concentration 19 μM) was added to RGK1 and RGM1 cells cultured in plastic dishes of 60 mm diameter (for AFM observation) or glass-bottom dishes of 35 mm diameter (for super-resolution observation), and the cells were incubated for 24 h in at 37 °C with a CO_2_ concentration of 5%. This PLP concentration is not toxic [15]. In a previous experiment in which the incubation time was varied [15], it was confirmed that the PLP concentration does not saturate in 3 h but saturates in 12 h. In this study, we incubated the cells for 24 h so that the amount of sensitizer entering the cells reached saturation.

**ER and mitochondria staining.** ER-Tracker™ Green (Invitrogen™ Thermo Fisher Scientific Inc., MA, USA) and MitoTracker™ Orange CMTMRos (Invitrogen™ Thermo Fisher Scientific Inc., MA, USA) were used for the fluorescence staining of the ER and mitochondria, respectively. PLP-added RGK1 and RGM1 cells were cultured in glass-bottom dishes of 35 mm diameter for 24 h in an incubator at 37 °C with a CO_2_ concentration of 5%. Next, 0.5 μL of 1 mM ER Tracker DMSO solution and 0.5 μL of 1 mM MitoTracker DMSO solution were added to 2 mL of MSF buffer solutions (5.4 mM KCl, 136.9 mM NaCl, 8.3 mM glucose, 0.44 mM KH_2_PO_4_, 0.33 mM Na_2_HPO_4_, 10.1 mM HEPES, 1 mM MgCl_6_ 6H_2_O, and 1 mM CaCl_2_ 2H_2_O) [31]. Then, this solution was replaced with the medium in the dish and incubated for 10 min. Finally, the staining solution in the dish was replaced with the medium, and the sample was observed under a super-resolution microscope.

**Actin filaments staining.** SPY-555 (Cytoskeleton Inc., CO, USA) was dissolved in 50 μL of anhydrous DMSO (anhydrous dimethyl sulfoxide), 2 μL of which was added to the cultured PLP-added RGK1 and RGM1 cells in glass-bottom dishes of 35 mm diameter. Then, the sample was incubated at 37 °C for 60 min. Finally, the staining solution in the dish was replaced with the medium, and the sample was observed under a super-resolution microscope.

**PDT and time-lapse observations.** Time-lapse observations were performed with an IX83 microscope system (Evident Corp., Tokyo, Japan) equipped with a stage-top incubator (Tokai Hit Co., Ltd., Fujinomiya-shi, Japan). A super-resolution observation unit (CSU-W1 SoRa: Yokogawa Electric Corp., Tokyo, Japan) was installed in the IX83 system and used to observe sites where PLP accumulated. For the light irradiation of PDT, we used a 640 nm semiconductor laser in the CSU-W1 SoRa system, where the sample was irradiated for 1 min at 439 mW/cm^2^.

**AFM observation.** AFM observation was performed by setting the AFM system (MFP-3D-BIO: Oxford Instruments plc, Tubney Woods, UK) on a microscope (IX71: Evident Corp., Tokyo, Japan) so that the elastic modulus of the same cells could be measured before and after light irradiation. After AFM observation using the IX71 system, the sample was moved to the stage top incubator placed on an IX83 system, irradiated with light, and then returned to the IX71 system. The light source used to illuminate the samples was a mercury white light (U-HG LGPS: Evident Corp., Tokyo, Japan) with a 635–675 nm bandpass filter. The same cells found by phase-contrast observation were observed by AFM. The elastic modulus was estimated by fitting the force curves, which were measured at each point of the cell during the approach of the AFM tip (Biolever BL-AC40TS-C2 cantilever: Evident Corp., Tokyo, Japan), with the Hertz contact mechanical model [14,29,32]. The force curves were measured in a region of 100 μm × 100 μm with a grid of 64 points × 64 points, used as an elastic modulus map, and the local elastic modulus was obtained from the force curve at each point using Hertz’s equation.

## 3. Results and Discussions

**Figure 3:**
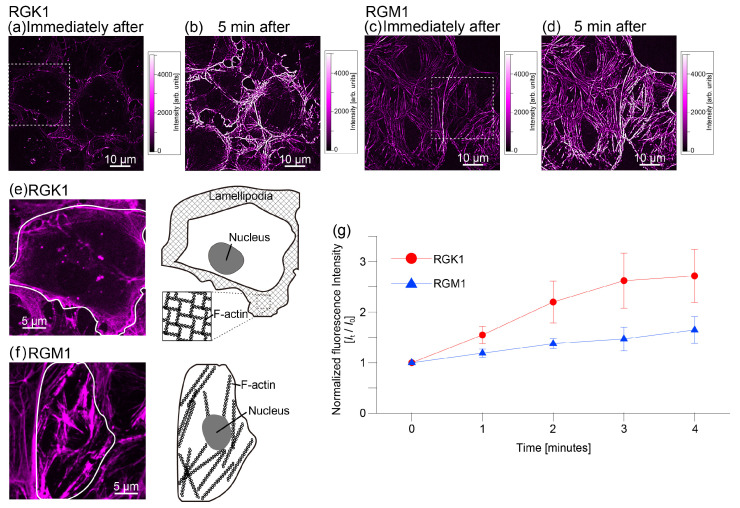
(**a**) Fluorescence images of actin filaments in RGK1 observed by super-resolution confocal microscopy immediately after 1 min of light irradiation and (**b**) 5 min later. (**c**) Fluorescence images of actin filaments in RGM1 observed by super-resolution confocal microscopy immediately after 1 min of light irradiation and (**d**) 5 min later. (**e**) Enlarged view of the area enclosed by the dashed line in (**a**), and a schematic diagram of one cell enclosed by a solid line. (**f**) Enlarged view of the area enclosed by the dashed line in (**b**), and a schematic diagram of one cell enclosed by a solid line. All fluorescence images are pseudo-colored in magenta. (**a**–**d**) were measured with a constant exposure time (300 ms) to compare the light intensity dependence of the processes, and the contrast in (**e**,**f**) was adjusted to highlight the state of the actin filaments. (**g**) Fluorescence intensity of actin filaments in the entire image immediately after light irradiation is set to 1, and the fluorescence intensity over time is shown.

Figure 3a–d show fluorescence images of actin filaments observed with super-resolution confocal microscopy for RGK1 and RGM1 immediately after 1 min of light irradiation and 5 min later, respectively. Figure 3e,f show enlarged views of Figure 3a and Figure 3b, respectively. The actin filaments in RGK1 are band-like, localized at the cell periphery, with few long, linear filaments. These features align with the characteristics of cancer cells, which display well-developed filopodia and lamellipodia [33,34,35]. Lamellipodia are thin, membranous structures at the cell periphery with a network of actin filaments [33,34,35,36,37]. Filopodia are slender cytoplasmic protrusions composed of actin filaments, extending beyond the leading edge of lamellipodia in migrating cells [33,34,35,36,37].

On the other hand, in RGM1 cells, no structures or indications of pseudopodium formation were observed, and many long, linear actin filaments were seen. In both RGK1 and RGM1 cells, an increase in the fluorescence intensity of actin filaments was observed from immediately after light irradiation to 5 min later. This indicates that light irradiation promotes actin filament production, clearly demonstrating this process. Figure 3g shows the changes in the fluorescence intensity of actin filaments over time, starting immediately after 1 min of light irradiation. Setting the initial fluorescence intensity immediately after irradiation to 1, RGK1 showed a 2.62-fold increase after 3 min, with no further increase thereafter. In contrast, in RGM1, the fluorescence intensity gradually increased and reached approximately 1.47 times the initial intensity after 3 min of light irradiation.

As noted above, ROS generated through PDT activate RhoA, leading to increased actin filament production. Higher ROS levels result in greater actin filament formation. Since RGK1 takes up more PLP than RGM1 [13], it likely produces more ROS, resulting in increased actin filament production and higher fluorescence intensity. Furthermore, since the amount of fluorescent probe (SPY-555) taken up by cells remains constant and actin is not newly synthesized within a few minutes, it is likely that after 4 min, the available SPY-555 and actin are fully utilized, leading to no further increase in fluorescence intensity.

**Figure 4:**
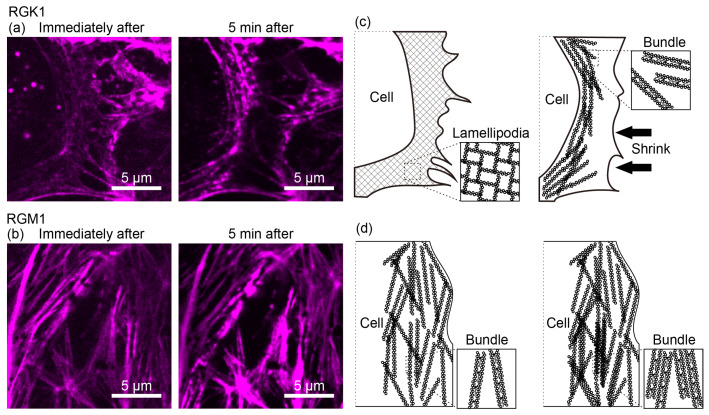
Fluorescence images of actin filaments in (**a**) RGK1 and (**b**) RGM1 cells observed by super-resolution confocal microscopy immediately after 1 min light irradiation and 5 min later. All fluorescence images are pseudo-colored in magenta, and the contrast is adjusted to highlight the state of the actin filaments. (**c**) Schematic diagram of (**a**). (**d**) Schematic diagram of (**b**).

A closer observation of the edges in RGK1 cells (Figure 4a) shows that pseudopodia observed immediately after light irradiation contracted and bundled within 5 min. In contrast, in RGM1 (Figure 4b), fluorescence intensity increased, but no significant shape changes occurred in the linear actin filaments. Figure 4c,d are schematics of Figure 4a,b. In both RGK1 and RGM1, actin filament production increased following PDT with PLP administration, but the increase was more pronounced in RGK1. Moreover, in RGK1 cells, the activation of myosin light chains likely caused the network-like actin filaments to bundle. In RGM1 cells, although actin filament production increased, it was lower than that in RGK1 cells, and minimal myosin light chain activation resulted in no cell contraction. Additionally, only slight bundling was observed in actin filaments, with no significant shape change.

**Figure 5:**
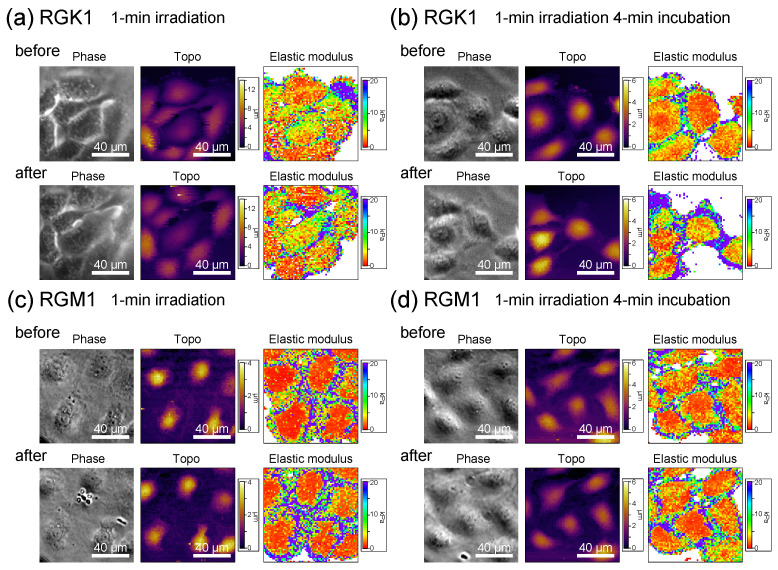
Phase contrast images, topography images, and elastic modulus maps of RGK1 and RGM1 cells with added PLP. (**a**,**c**) immediately after 1 min light irradiation. (**b**,**d**) after 1 min light irradiation and following 4 min standing in an incubator at 37 °C with 5% CO_2_ concentration. Force curves were measured at 64 × 64 points in an area of 100 μm × 100 μm. The topo images were obtained by mapping the height when a force of 200 pN was measured.

The fluorescence analysis of actin filament bundling induced by activated myosin light chains makes it difficult to characterize the increase in the quantity of stress fibers, because of the additional production of ROS throughout the process. To assess stress fiber formation, we measured the cells’ elastic modulus, which reflects changes in the cytoskeleton. The elastic modulus was measured mechanically by AFM [14,29]. The local elastic moduli of RGK1 and RGM1 cells treated with PLP were measured using the same cells before and after light irradiation, and the results were compared. Figure 5 shows phase contrast images, topography images, and elastic modulus maps of RGK1 and RGM1 cells. In Figure 5a, the elastic modulus of RGK1 cells immediately after 1 min light irradiation showed little change around its nuclei or edges. However, after 4 min incubation following 1 min light irradiation (Figure 5b), the elastic modulus at the cell edges significantly increased. In contrast, there was no significant increase in the elastic modulus of RGM1 cells, either immediately after 1 min light irradiation (Figure 5c) or after a 4 min incubation following 1 min of light irradiation (Figure 5d).

**Figure 6:**
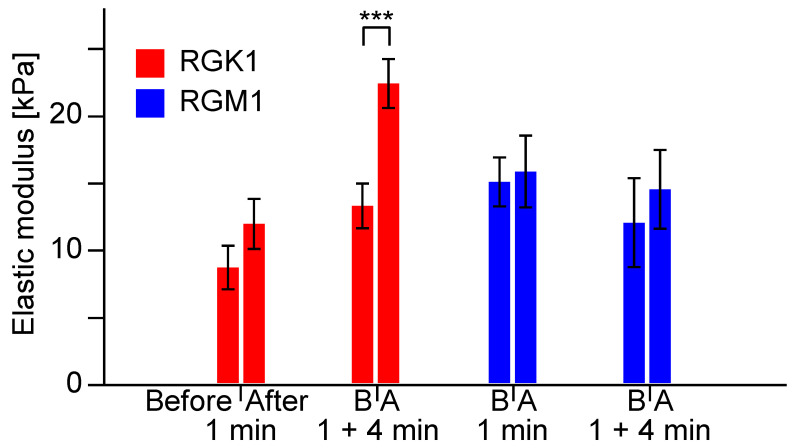
The average elastic moduli of RGK1 and RGM1 cells immediately after 1 min light irradiation and after 4 min incubation following the irradiation. “Before/B” refers to before light irradiation, and “After/A” refers to after light irradiation. *** *p* < 0.01.

Figure 6 shows the average elastic moduli of RGK1 and RGM1 cells immediately after 1 min light irradiation and after 4 min incubation following the irradiation. The average elastic moduli of RGK1 cells before and after 1 min of irradiation were 8.58 kPa and 11.8 kPa, and after 4 min incubation following irradiation, the average elastic moduli increased to 13.2 kPa and 22.3 kPa, respectively. The average elastic modulus increased approximately 1.4 times immediately after irradiation and 1.7 times after 4 min incubation.

On the other hand, the average elastic moduli of RGM1 cells before and after 1 min irradiation were 14.9 kPa and 15.7 kPa, and after 5 min incubation following irradiation, the average elastic moduli increased to 11.9 kPa and 14.4 kPa, respectively. The average elastic moduli of RGM1 cells increased approximately 1.05 times immediately after irradiation and 1.2 times after 4 min incubation; both increases are smaller than those of RGK1 cells.

**Figure 7:**
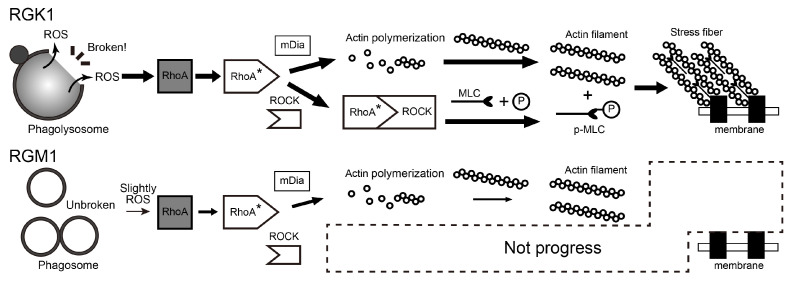
Schematic diagram of the mechanism by which RGK1 and RGM1 produce stress fibers. RhoA, Ras homolog family member A; RhoA*, activated RhoA; mDia, mammalian diaphanous-related formin; ROCK, RhoA–Rho-associated protein kinase; MLC, myosin light chain; and pMLC, phospho-myosin light chain. The thickness of the arrows in the processes for RGM1 and RGK1 represents the degree of involvement of each process in the mechanism.

On the basis of these results, we discuss the different stress fiber formation mechanisms in RGK1 and RGM1 cells. Figure 7 shows a schematic of stress fiber production mechanisms in RGK1 and RGM1 cells. Stress fiber formation via PDT begins with singlet oxygen, which is converted into other ROS such as OH^−^ or H_2_O_2_ [38,39,40,41]. These ROS activate RhoA [26,27,38,42]. The activated RhoA acts on mammalian diaphanous-related formin (mDia) to promote actin polymerization and binds to Rho-associated protein kinase (ROCK) to induce myosin light chain phosphorylation [26,27,42,43,44]. The phosphorylated myosin light chains bind to actin filaments, bundling them into stress fibers. These fibers generate tension by linking to focal adhesions on the cell membrane, affecting the cell elastic modulus [14,29].

In RGK1 cells, during the 1 min irradiation, phagolysosomes remain intact, preventing ROS leakage; thus, only singlet oxygen from PLP activates stress fiber production. The small amount of ROS generated at this stage induces minimal stress fiber formation and a slight increase in elastic modulus. After 1 min of irradiation, phagolysosomes are destroyed, allowing leaked ROS to enhance the role of ROS produced from PLP, thereby activating further stress fiber formation. In the blood flow-blocking mechanism of PDT, it takes approximately 5 min for ROS to activate RhoA and myosin light chains [14,26,29]. Thus, no immediate change in elastic modulus occurs after light irradiation; stress fibers form about 5 min later, increasing the elastic modulus of cells.

In RGM1 cells, the increase in actin filament fluorescence intensity shown in Figure 3e is smaller than that in RGK1 but still approximately 1.5 times. However, the elastic modulus showed no significant change after 4 min incubation following 1 min light irradiation. RGM1 cells likely contain few or no phagolysosomes, or their phagosomes remain intact, preventing ROS leakage. Most ROS generated by PDT are likely singlet oxygen from PLP. As a result, the mechanism of the increase in the formation of actin filaments is activated, but not that of myosin light chains, preventing stress fiber formation and significant increases in elastic modulus.

## 4. Conclusions

In conclusion, in this study, we observed stress fiber production induced by PDT with PLP, using super-resolution confocal microscopy to observe actin filaments and AFM to measure cell elastic modulus. We identified different intracellular mechanisms of stress fiber formation between RGK1 and RGM1 cells. In RGK1 cells, ROS generated from PLP destroy phagolysosomes, and the leaked ROS further enhances stress fiber production. In RGM1 cells, phagosomes are not destroyed by PDT, so only ROS from PLP activate stress fiber production. However, since the amount of ROS from PLP is small, actin filaments are produced but do not activate myosin light chains, resulting in limited stress fiber formation.

## Data Availability

Data is contained within the article.

## References

[1] Sang N., Qi Y., Nishimura S., Miyako E. (2024). Biomimetic Functional Nanocomplexes for Photothermal Cancer Chemoimmunotheranostics. Small Sci..

[2] Tanita K., Koseki Y., Kumar S., Taemaitree F., Mizutani A., Nakatsuji H., Suzuki R., Dao A.T.N., Fujishima F., Tada H. (2024). Carrier-Free Nano-Prodrugs for Minimally Invasive Cancer Therapy. Nanoscale.

[3] Luo M., Yukawa H., Sato K., Tozawa M., Tokunaga M., Kameyama T., Torimoto T., Baba Y. (2022). Multifunctional Magnetic CuS/Gd2O3 Nanoparticles for Fluorescence/Magnetic Resonance Bimodal Imaging-Guided Photothermal-Intensified Chemodynamic Synergetic Therapy of Targeted Tumors. ACS Appl. Mater. Interfaces.

[4] Cheng W., Nie J., Xu L., Liang C., Peng Y., Liu G., Wang T., Mei L., Huang L., Zeng X. (2017). pH-Sensitive Delivery Vehicle Based on Folic Acid-Conjugated Polydopamine-Modified Mesoporous Silica Nanoparticles for Targeted Cancer Therapy. ACS Appl. Mater. Interfaces.

[5] Doi M., Tanaka H., Ohoto T., Miura N., Sakurai Y., Hatakeyama H., Akita H. (2023). Reactivation of Anticancer Immunity by Resetting Interorgan Crosstalk in Immune-Suppressive Cells with a Nanoparticulated Anti-Inflammatory Drug. Small.

[6] Gao X., Zhang J., Huang Z., Zuo T., Lu Q., Wu G., Shen Q. (2017). Reducing Interstitial Fluid Pressure and Inhibiting Pulmonary Metastasis of Breast Cancer by Gelatin Modified Cationic Lipid Nanoparticles. ACS Appl. Mater. Interfaces.

[7] He C., Duan X., Guo N., Chan C., Poon C., Weichselbaum R.R., Lin W. (2016). Core-Shell Nanoscale Coordination Polymers Combine Chemotherapy and Photodynamic Therapy to Potentiate Checkpoint Blockade Cancer Immunotherapy. Nat. Commun..

[8] Nichols J.W., Bae Y.H. (2014). EPR: Evidence and Fallacy. J. Control. Release.

[9] Danhier F. (2016). To Exploit the Tumor Microenvironment: Since the EPR Effect Fails in the Clinic, What Is the Future of Nanomedicine?. J. Control. Release.

[10] Lovell J.F., Jin C.S., Huynh E., Jin H., Kim C., Rubinstein J.L., Chan W.C.W., Cao W., Wang L.V., Zheng G. (2011). Porphysome Nanovesicles Generated by Porphyrin Bilayers for Use as Multimodal Biophotonic Contrast Agents. Nat. Mater..

[11] Cui L., Lin Q., Jin C.S., Jiang W., Huang H., Ding L., Muhanna N., Irish J.C., Wang F., Chen J. (2015). A PEGylation-Free Biomimetic Porphyrin Nanoplatform for Personalized Cancer Theranostics. ACS Nano.

[12] Lou J., Aragaki M., Bernards N., Kinoshita T., Mo J., Motooka Y., Ishiwata T., Gregor A., Chee T., Chen Z. (2021). Repeated Porphyrin Lipoprotein-Based Photodynamic Therapy Controls Distant Disease in Mouse Mesothelioma via the Abscopal Effect. Nanophotonics.

[13] Taninaka A., Kurokawa H., Kamiyanagi M., Ochiai T., Arashida Y., Takeuchi O., Matsui H., Shigekawa H. (2023). Polphylipoprotein-Induced Autophagy Mechanism with High Performance in Photodynamic Therapy. Commun. Biol..

[14] Kamiyanagi M., Taninaka A., Ugajin S., Nagoshi Y., Kurokawa H., Ochiai T., Arashida Y., Takeuchi O., Matsui H., Shigekawa H. (2022). Cell-Level Analysis Visualizing Photodynamic Therapy with Porphylipoprotein and Talaporphyrin Sodium. Int. J. Mol. Sci..

[15] Kurokawa H., Ito H., Matsui H. (2021). Porphylipoprotein Accumulation and Porphylipoprotein Photodynamic Therapy Effects Involving Cancer Cell-Specific Cytotoxicity. Int. J. Mol. Sci..

[16] Jiang M., Esteve-Rudd J., Lopes V.S., Diemer T., Lillo C., Rump A., Williams D.S. (2015). Microtubule Motors Transport Phagosomes in the RPE, and Lack of KLC1 Leads to AMD-like Pathogenesis. J. Cell Biol..

[17] Blocker A., Severin F.F., Habermann A., Hyman A.A., Griffiths G., Burkhardt J.K. (1996). Microtubule-Associated Protein-Dependent Binding of Phagosomes to Microtubules (∗). J. Biol. Chem..

[18] Blocker A., Griffiths G., Olivo J.-C., Hyman A.A., Severin F.F. (1998). A Role for Microtubule Dynamics in Phagosome Movement. J. Cell Sci..

[19] Fu M., Holzbaur E.L.F. (2013). JIP1 Regulates the Directionality of APP Axonal Transport by Coordinating Kinesin and Dynein Motors. J. Cell Biol..

[20] Horiuchi D., Collins C.A., Bhat P., Barkus R.V., DiAntonio A., Saxton W.M. (2007). Control of a Kinesin-Cargo Linkage Mechanism by JNK Pathway Kinases. Curr. Biol..

[21] Kant S., Standen C.L., Morel C., Jung D.Y., Kim J.K., Swat W., Flavell R.A., Davis R.J. (2017). A Protein Scaffold Coordinates SRC-Mediated JNK Activation in Response to Metabolic Stress. Cell Rep..

[22] Nihalani D., Wong H.N., Holzman L.B. (2003). Recruitment of JNK to JIP1 and JNK-Dependent JIP1 Phosphorylation Regulates JNK Module Dynamics and Activation*. J. Biol. Chem..

[23] Dougherty T.J., Gomer C.J., Henderson B.W., Jori G., Kessel D., Korbelik M., Moan J., Peng Q. (1998). Photodynamic Therapy. JNCI J. Natl. Cancer Inst..

[24] Oleinick N.L., Morris R.L., Belichenko I. (2002). The Role of Apoptosis in Response to Photodynamic Therapy: What, Where, Why, and How. Photochem. Photobiol. Sci..

[25] Saito A., Nagao T., Minamitani H., Iino T., Yamamoto T., Aizawa K. Vascular Shut down Effect on the Microcirculation in Photodynamic Therapy Using Zinc Coproporphyrin. Proceedings of the 19th Annual International Conference of the IEEE Engineering in Medicine and Biology Society. “Magnificent Milestones and Emerging Opportunities in Medical Engineering” (Cat. No.97CH36136).

[26] Suzuki T., Tanaka M., Sasaki M., Ichikawa H., Nishie H., Kataoka H. (2020). Vascular Shutdown by Photodynamic Therapy Using Talaporfin Sodium. Cancers.

[27] Cavin S., Riedel T., Rosskopfova P., Gonzalez M., Baldini G., Zellweger M., Wagnières G., Dyson P.J., Ris H.-B., Krueger T. (2019). Vascular-Targeted Low Dose Photodynamic Therapy Stabilizes Tumor Vessels by Modulating Pericyte Contractility. Lasers Surg. Med..

[28] Karwicka M., Pucelik B., Gonet M., Elas M., Dąbrowski J.M. (2019). Effects of Photodynamic Therapy with Redaporfin on Tumor Oxygenation and Blood Flow in a Lung Cancer Mouse Model. Sci. Rep..

[29] Taninaka A., Ugajin S., Kurokawa H., Nagoshi Y., Kamiyanagi M., Takeuchi O., Matsui H., Shigekawa H. (2022). Direct Analysis of the Actin-Filament Formation Effect in Photodynamic Therapy. RSC Adv..

[30] Shimokawa O., Matsui H., Nagano Y., Kaneko T., Shibahara T., Nakahara A., Hyodo I., Yanaka A., Majima H.J., Nakamura Y. (2008). Neoplastic Transformation and Induction of H+,K+-Adenosine Triphosphatase by N-Methyl-N′-Nitro-N-Nitrosoguanidine in the Gastric Epithelial RGM-1 Cell Line. Vitr. Cell. Dev. Biol. Anim..

[31] Kurokawa H., Taninaka A., Shigekawa H., Matsui H. (2021). Dabigatran Etexilate Induces Cytotoxicity in Rat Gastric Epithelial Cell Line via Mitochondrial Reactive Oxygen Species Production. Cells.

[32] Sneddon I.N. (1965). The Relation between Load and Penetration in the Axisymmetric Boussinesq Problem for a Punch of Arbitrary Profile. Int. J. Eng. Sci..

[33] Sahai E. (2005). Mechanisms of Cancer Cell Invasion. Curr. Opin. Genet. Dev..

[34] Parri M., Chiarugi P. (2010). Rac and Rho GTPases in Cancer Cell Motility Control. Cell Commun. Signal..

[35] Sanz-Moreno V., Marshall C.J. (2010). The Plasticity of Cytoskeletal Dynamics Underlying Neoplastic Cell Migration. Curr. Opin. Cell Biol..

[36] Medeiros N.A., Burnette D.T., Forscher P. (2006). Myosin II Functions in Actin-Bundle Turnover in Neuronal Growth Cones. Nat. Cell Biol..

[37] Mattila P.K., Lappalainen P. (2008). Filopodia: Molecular Architecture and Cellular Functions. Nat. Rev. Mol. Cell Biol..

[38] Sharman W.M., Allen C.M., van Lier J.E. (2000). Role of Activated Oxygen Species in Photodynamic Therapy. Methods in Enzymology.

[39] Pass H.I. (1993). Photodynamic Therapy in Oncology: Mechanisms and Clinical Use. JNCI J. Natl. Cancer Inst..

[40] Foote C.S. (1991). Definition of type i and type ii photosensitized oxidation. Photochem. Photobiol..

[41] Ethirajan M., Chen Y., Joshi P., Pandey R.K. (2010). The Role of Porphyrin Chemistry in Tumor Imaging and Photodynamic Therapy. Chem. Soc. Rev..

[42] Kajimoto H., Hashimoto K., Bonnet S.N., Haromy A., Harry G., Moudgil R., Nakanishi T., Rebeyka I., Thébaud B., Michelakis E.D. (2007). Oxygen Activates the Rho/Rho-Kinase Pathway and Induces RhoB and ROCK-1 Expression in Human and Rabbit Ductus Arteriosus by Increasing Mitochondria-Derived Reactive Oxygen Species. Circulation.

[43] Wirth A. (2010). Rho Kinase and Hypertension. Biochim. Biophys. Acta (BBA) Mol. Basis Dis..

[44] Hartmann S., Ridley A.J., Lutz S. (2015). The Function of Rho-Associated Kinases ROCK1 and ROCK2 in the Pathogenesis of Cardiovascular Disease. Front. Pharmacol..

